# Merging Modular Molecular Design with High Throughput Screening of Cell Adhesion on Antimicrobial Supramolecular Biomaterials

**DOI:** 10.1002/marc.202300638

**Published:** 2024-04-13

**Authors:** Moniek G. J. Schmitz, Jasper G. M. Aarts, Laurence Burroughs, Phanikrishna Sudarsanam, Tim J. M. Kuijpers, Martijn Riool, Leonie de Boer, Xuan Xue, Dragan Bosnacki, Sebastian A. J. Zaat, Jan de Boer, Morgan R. Alexander, Patricia Y. W. Dankers

**Affiliations:** ^1^ Department of Biomedical Engineering Institute for Complex Molecular Systems (ICMS) Eindhoven University of Technology PO Box 513 Eindhoven 5600 MB The Netherlands; ^2^ School of Pharmacy University of Nottingham University Park Nottingham NG7 2RD UK; ^3^ Department of Medical Microbiology and Infection Prevention Amsterdam institute for Infection and Immunity Amsterdam University Medical Centers University of Amsterdam Amsterdam 1105 AZ The Netherlands; ^4^ Present address: Laboratory of Experimental Trauma Surgery, Department of Trauma Surgery University Hospital Regensburg Am Biopark 9 93053 Regensburg Germany

**Keywords:** antimicrobial, array, biomaterials, high throughput, screening, supramolecular, upy

## Abstract

A polymer microarray based on the supramolecular ureido‐pyrimidinone (UPy) moiety is fabricated to screen antimicrobial materials for their ability to support cell adhesion. UPy‐functionalized additives, either cell‐adhesive, antimicrobial or control peptides, are used, and investigated in different combinations at different concentrations, resulting in a library of 194 spots. These are characterized on composition and morphology to evaluate the microarray fabrication. Normal human dermal fibroblasts are cultured on the microarrays and cell adhesion to the spots is systematically analyzed. Results demonstrate enhanced cell adhesion on spots with combinations including the antimicrobial peptides. This study clearly proves the power of the high throughput approach in combination with supramolecular molecules, to screen additive libraries for desired biological response.

## Graphical abstract

Combinatorial high throughput screening using polymer microarrays has become an established method for discovering new biomaterials. This study creates a supramolecular ureido‐pyrimidinone (UPy) polymer microarray to screen antimicrobial materials for cell adhesion. Results show enhanced cell adhesion on spots containing (combinations of) antimicrobial peptides. This approach efficiently screens additive libraries for desired biological responses.

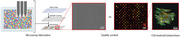

## 1 Introduction

Control over cell‐material interactions is essential for the performance of biomaterials and demands different material characteristics depending on the application. To this end, biomaterials often need to be multifunctional and require integration of multiple bioactive cues. The traditional method of biomaterial development starts with a specific biomaterial design, followed by thorough material characterization and extensive read‐out studies, which possibly results in a change of the biomaterial design to start all over again until the desired output is reached.^[^ ^1^, ^2^ ^]^ This is very labor‐intensive, time‐consuming and does not leave room for many test conditions and out‐of‐the‐box material discoveries. Many contemporary combinatorial and computational methods are applicable to biomaterials design, and can be utilized to accelerate the discovery of new biomaterials analogously, to their application to other fields such as drug discovery.^[^ ^3^, ^4^ ^]^ and chemical synthesis.^[^ ^5^ ^]^

Combinatorial high throughput screening has already been successfully used to discover new biomaterials and enables screening of big material libraries for specific biological demands.^[^ ^6^, ^7^, ^8^ ^]^ One way to perform high throughput screening of polymeric materials is via fabrication of microarrays. In a microarray hundreds or even thousands of individual material spots are spatially distributed on a glass slide. This allows to systematically investigate the interactions between unique material spots and a biological response.

In the first contact‐printed microarray published by Anderson, Levenberg and Langer, an in‐situ synthesis strategy was used to develop a microarray with 576 different combinations of 25 different monomers with a light‐activated radical initiator. First, different combinations of the monomer solutions were printed, followed by polymerization via ultra violet (UV) irradiation and finalized with solvent removal. The fabricated microarray facilitated high throughput screening of growth and differentiation of human embryonic stem cells.^[^ ^9^ ^]^ A year later, Anderson et al. published the fabrication of a microarray with different mixtures of pre‐synthesized biodegradable polyester polymers.^[^ ^10^ ^]^ and a similar approach was used with polyurethanes on agarose gel‐coated slides by Bradley and co‐workers in 2006.^[^ ^11^ ^]^ In the years that followed, large material libraries on polymer microarrays were screened and by the use of computer modelling and machine learning analyzed for specific biological responses, such as differentiation of human embryonic stem cells into cardiomyocytes,^[^ ^12^ ^]^ inducing macrophage polarization,^[^ ^13^ ^]^ and bacterial attachment prevention.^[^ ^14^, ^15^, ^16^ ^]^

Supramolecular polymers are particularly suited for biomaterial design because of their intrinsically dynamic nature and because they are highly tunable and modular.^[^ ^17^ ^]^ The modularity makes supramolecular materials ideal candidates for the design of high throughput microarray libraries, because bioactivity can easily be introduced in these materials via a simple mix‐and‐match approach. Functionalization of the bioactive molecule with a supramolecular molecule allows incorporation into the material via non‐covalent interactions. This way, many functionalities can easily be introduced, added in different combinations, in various concentrations and screened for the desired biological response.^[^ ^18^ ^]^

In our group, supramolecular biomaterials are based on 2‐ureido‐4[1H]pyrimidinone (UPy) moieties which self‐dimerize upon fourfold hydrogen bonding.^[^ ^19^, ^20^ ^]^ Stacking of the UPy dimers via π‐π interactions is stabilized by the presence of an additional urea group, and induces nanofiber formation in the lateral direction.^[^ ^21^, ^22^ ^]^ Functionalization with short pre‐polymers, such as polycaprolactone or polycarbonate, results in thermoplastic elastomeric materials.^[^ ^23^ ^]^ These UPy‐materials can be functionalized with bioactive compounds via a modular approach in which the UPy‐base material is mixed with UPy‐modified additives.^[^ ^24^, ^25^ ^]^ This has resulted in materials with a variety of characteristics such as materials with anti‐fouling properties,^[^ ^26^, ^27^ ^]^ cell‐adhesive cues,^[^ ^28^, ^29^ ^]^ cytokine complexation,^[^ ^30^ ^]^ Notch‐activation,^[^ ^31^ ^]^ and antimicrobial activity.^[^ ^32^ ^]^

**Figure 1:**
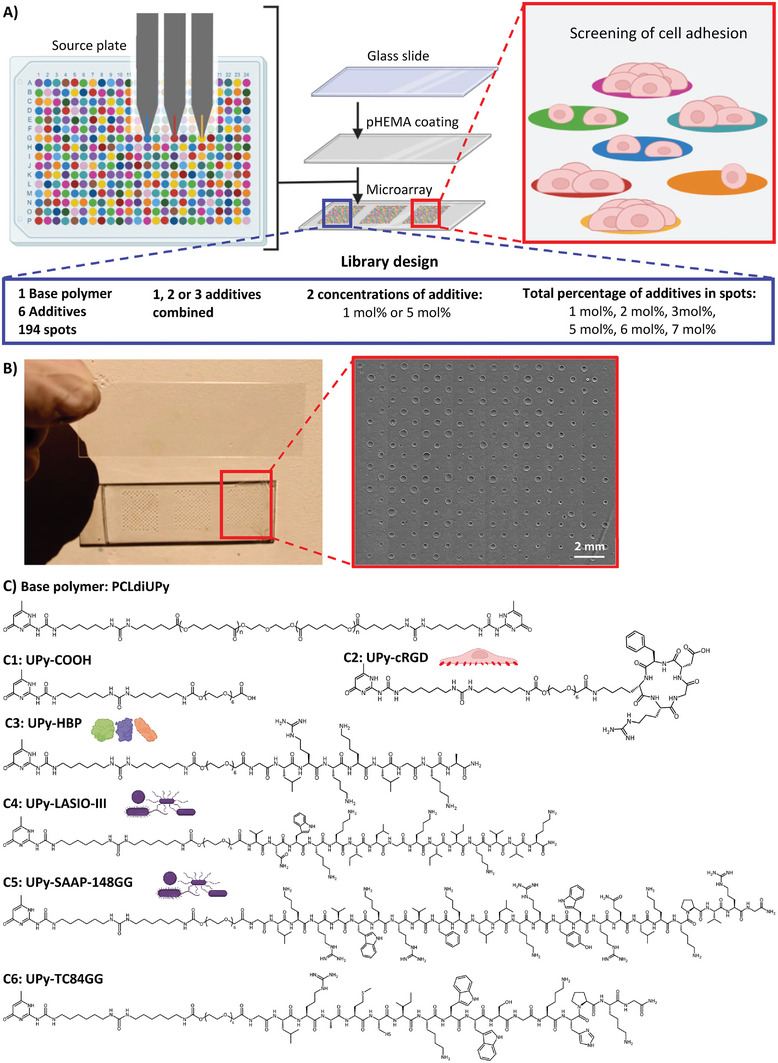
Overview of the supramolecular microarray design used for NHDF adhesion screening. A) Schematic representation of the microarray production via contact printing of supramolecular solutions and library design. B) Image of complete microarray and brightfield phase contrast image of one replicate of the polymer microarray. C) Molecular structures of the molecules in the microarray formulations. From top to bottom: PCLdiUPy (base polymer; C), UPy‐COOH (control; C1), UPy‐HBP (binding of growth factors; C2), UPy‐cRGD (cell adhesive; C3), UPy‐LASIO‐III (antimicrobial; C4), UPy‐SAAP‐148GG (antimicrobial; C5), UPy‐TC84GG (control; C6).

In this study, we developed a polymer microarray, based on the supramolecular UPy chemistry, to screen antimicrobial materials for normal human dermal fibroblast (NHDF) adhesion to develop a biomaterial which on one hand is antimicrobial and on the other hand promotes cell adhesion (**Figure** **1**A). To this end 6 different UPy‐functionalized additives were investigated, that have been individually studied before: a neutral additive, UPy‐COOH (C1);^[^ ^33^ ^]^ two UPy‐functionalized cell adhesive peptides, UPy‐cRGD^[^ ^34^ ^]^ (C2) and UPy‐heparin binding peptide^[^ ^35^ ^]^ (UPy‐HBP; C3). The other three additives were synthesized with the goal to act as UPy‐functionalized antimicrobial peptides (UPy‐AMPs). Zaccaria et al., showed that the lowest critical concentration in solution at which 99.9% of the bacteria are killed (LC99.9) for the UPy‐LASIO‐III (C4) additive ranged between 3.7 and 7.5 µm for *Escherichia coli* ATCC 8937, methicillin‐sensitive (MSSA; RN 422039) and methicillin‐resistant (MRSA; AMC 20140, 41) strains of *Staphylococcus aureus*.^[^ ^32^ ^]^ A similar concentration range of 1.25–5 µM for UPy‐LASIO‐III was shown for *S. aureus* JAR060131 and *E. coli* 8735 by Song and coworkers.^[^ ^36^ ^]^ When UPy‐LASIO‐III was incorporated as additive (1 and 4 mol%) in PCLdiUPy cast surfaces, the antimicrobial activity, assessed with a Japanese Industrial Standard test (JIS Z 2801:2000),^[^ ^37^ ^]^ showed a significant log reduction in bacterial growth for all three evaluated strains.^[^ ^32^ ^]^ The second UPy‐AMP, Upy‐SAAP‐148GG (C5), showed a LC99.9 between 0.93 and 1.88 µM for bacterial strains *S. aureus* JAR060131, the multidrug resistant strains *S. aureus* LUH14616 and *Acinetobacter baumannii* RUH875, and an extended spectrum beta‐lactamase (ESBL)‐producing *E. coli* strain. When mixed with PCLdiUPy and cast as a surface 5mol% of UPy‐SAAP‐148GG showed complete killing for all bacterial strains.^[^ ^38^ ^]^ Last, UPy‐TC84GG, a UPy‐AMP which has lost its antimicrobial activity, was used as control (C6). As base polymer, a telechelic UPy‐functionalized polycaprolactone with a M_n_ of 2 kDa (i.e. PCLdiUPy; C) was used (Figure 1C).^[^ ^24^, ^39^ ^]^ Different combinations of the 6 UPy additives were designed, allowing investigation of cell adhesion to the antimicrobial peptides and cell adhesive peptides separately, and to study possible synergistic effects on cell adhesion of the combination of both the UPy‐AMPs and the UPy functionalized cell adhesive peptides. To facilitate screening of the optimum combination of peptides, a microarray library was designed with 194 combinations of the 6 additives. In each combination 1, 2 or a maximum of 3 additives were combined, and additives were present in either 1 or 5 mol%, with a limit of 7 mol% additive in total. This resulted in combinations of additives with either 1, 2, 3, 5, 6, or 7 mol% additive in the material (Figure 1A). The library was analyzed with time‐of‐flight secondary ion mass spectrometry (ToF‐SIMS) for quality control and atomic force microscopy (AFM) to assess the spot morphology. The microarray (Figure 1B) was tested for NHDF adhesion and the effect of each additive or a combination of additives on cell adhesion was systematically analyzed.

## 2 Results and Discussion

### 2.1 Microarray Development and Characterization

A supramolecular polymer microarray, consisting of 3 replicates of 194 spots, was fabricated via automated contact printing from dimethylsulfoxide (DMSO). All spots consisted of the major component (93–100 mol%) PCLdiUPy base polymer, mixed with either 1 or a combination of 2 or 3 UPy‐additives, at either 1 or 5 mol% with a maximum of 7 mol% UPy additives. The spots were printed on a poly(hydroxyethyl methacrylate) (pHEMA) coated epoxy silanized microscope slide to facilitate printing and prevent cell adhesion outside the spots.^[^ ^9^ ^]^ One microarray was submerged in phosphate‐buffered saline (PBS) for 2 weeks at 37 °C, and the spots remained firmly attached to the pHEMA glass slides after this incubation period. The size of the spots was analyzed using bright field microscopy (Figure 1B). Overall, a large distribution of the spot size was noted, between 0.05 and 0.33 mm^2^. The reason for this is twofold. First, the size of all replicates differs significantly, probably because the microscope slide was not completely leveled during printing (Figure S3B, Supporting Information). Second, variations were found between spots where certain types of additives were present (Figure S3D, Supporting Information). For instance, the addition of 1 mol% UPy‐COOH (C1) results in significantly smaller spots when compared to the addition of 5 mol% UPy‐COOH (C1). Variations in spot size are caused by the different hydrophilic characters of the additives causing the spots to spread better. To correct for the difference in spot size, the cell count was normalized to the spot size.

**Figure 2:**
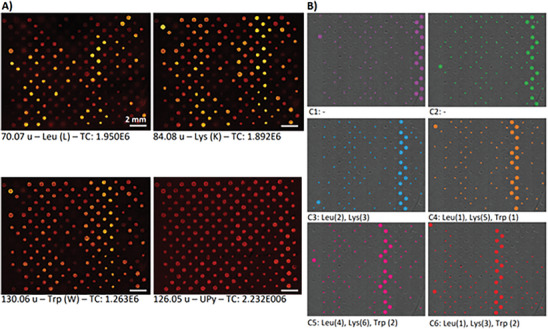
ToF‐SIMS ion images from one replicate area of the microarray and schematic overview of the presence of C1‐C6 within the library. A) ToF‐SIMS ion images for the side chains of leucine (m/z 70.07), lysine (m/z 84.08), tryptophan (m/z 130.06) and UPy (m/z 126.05). Scale bars represent 2 mm. B) Schematic overview of the presence of C1‐C6 in the library. Between brackets the prevalence of the amino acids in the peptide is shown. A larger colored spot represents 5 mol% of the additive and a small colored spot 1 mol%.

Automated ToF‐SIMS was performed as quality control to characterize the surface of the microarray and check the material composition. Graphs of the ion intensity from one replicate for representative SIMS ions were correlated to the presence of these molecules in the spots, in which a small colored spot represented 1 mol% and a big colored spot 5 mol% (**Figure** **2**A,B). More specifically, the ions of the amino acid side chains of leucine (m/z 70.07),^[^ ^40^ ^]^ lysine (m/z 84.08)^[^ ^40^ ^]^ tryptophan (m/z 130.06),^[^ ^40^ ^]^ and the UPy (m/z 126.05)^[^ ^41^ ^]^ ion were selected. The ion for UPy showed an equal intensity for all spots. The ions representing leucine, lysine and tryptophan were detected in patterns corresponding with the design. Besides this, all spots were found to be confined in the correct pattern and no artifacts were observed such as a coffee ring effect which can result in empty donut‐shaped spots. For all four ions depicted (Figure 2A), some spots show an inhomogeneous distribution of the ion within the spot, which is an indication for phase separation of the molecules within the spot.

For most studies in which UPy‐polymers with UPy‐additives are cast on glass substrates, hexafluoroisopropanol (HFIP) is used as solvent since it quickly evaporates, yields a fiber morphology.^[^ ^21^, ^42^ ^]^ and smooth and elastic film.^[^ ^43^ ^]^ However, to be able to contact print the microarray and prevent evaporation during the printing process, all compounds were dissolved in DMSO and dried under vacuum. Since the morphology and phase separation of solution cast UPy‐polymer films depends greatly on the solvent that is used for casting, AFM measurements were performed before microarray fabrication.^[^ ^42^ ^]^ Peptide incorporation and fiber formation of 2 µL dropcast PCLdiUPy with 5 mol% UPy‐cRGD films from HFIP were compared to films cast from DMSO. Phase images showed a fiber morphology for both HFIP and DMSO cast PCLdiUPy films, with the DMSO cast fibers showing greater bundling and increased surface roughness (Figure S1, Supporting Information). Addition of 5 mol% UPy‐cRGD (C2) showed typical phase separation for casting from HFIP as noted before with 4 mol% UPy‐cRGD surfaces,^[^ ^34^ ^]^ but no phase separation for DMSO. The UPy‐cRGD was completely mixed into the PCLdiUPy base polymer, probably due to the slower evaporation. This may influence the bioactivity of the UPy‐peptides and must be kept in mind during upscaling of the high throughput screening hits.

### 2.2 Eukaryotic Cell Adhesion to Microarray Spots

**Figure 3:**
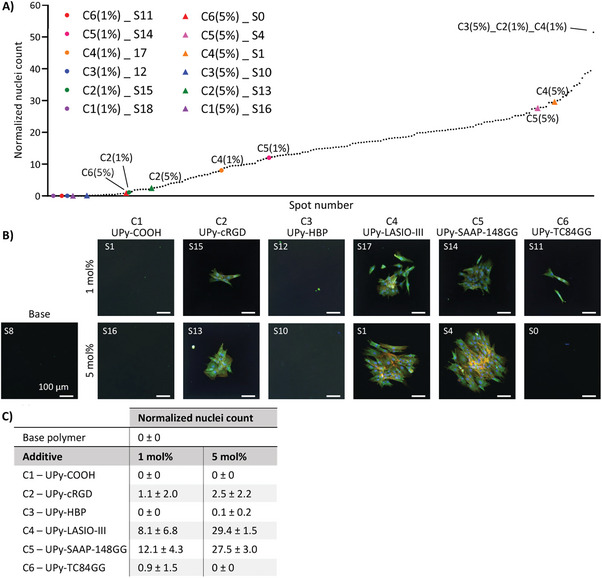
NHDF cell adhesion on microarray spots. A) Rank plot of all microarray spots of the averaged, normalized nuclei count of NHDFs with the single additive spots highlighted. B) Cell morphology of corresponding spots with in blue the nucleus, orange the cytoskeleton and green α‐SMA. Scale bars represent 100 µm. C) Averaged and normalized nuclei count for the base polymer spot (C0) and spots with only one additive (C1‐C6) at either 1 or 5 mol%.

The ability of the different material spots to support cell adhesion was investigated. NHDFs were cultured on the microarray for 48 h. The data showed a spread in the normalized nuclei count from 0.0 to 51.4 (**Figure** **3**A). Upon correlation of the number of nuclei to the spot size no trend was observed, indicating that a bigger spot size, did not automatically result in more adhered nuclei (Figure S2, Supporting Information). Therefore the effect of the different spot sizes and thereby surface topologies on cell adhesion could be neglected compared to the effect of material composition. Fluorescence microscopy images of the corresponding spots showed cells with a spreading morphology that fully covered the area of the spots containing 5 mol% of the AMPs UPy‐LASIO‐III (C4) and UPy‐SAAP‐148GG (C5) (Figure 3B). Spots that consist of only the base polymer PCLdiUPy did not have any adhering cells. This is no surprise, since PCLdiUPy generally requires a bioactive component for proper cell adhesion.^[^ ^39^ ^]^ Also UPy‐COOH (C1), UPy‐HBP (C3) and UPy‐TC84GG (C6) did not show any cell adhesion. Addition of UPy‐cRGD (C2), did result in a few adhering cells, but the majority of the spot area was empty. The cell adhesion here is lower than reported previously, but may be explained by several factors, such as cell type used,^[^ ^38^ ^]^ the oligo(ethylene glycol) spacer length,^[^ ^34^ ^]^ material formulation,^[^ ^41^ ^]^ and the solvent used for casting. Only spots with the AMPs (i.e. compound C4 or C5) showed high cell adhesion, with an increase in cell number for 5 mol% compared to 1 mol% of the compound (Figure 3C). Since both C4 and C5 contain many charged amino acids, like lysine and arginine, this may have caused non‐covalent interactions between the material and proteins in the fetal bovine serum (FBS). This would eventually lead to more cells adhering to the material surface.

**Figure 4:**
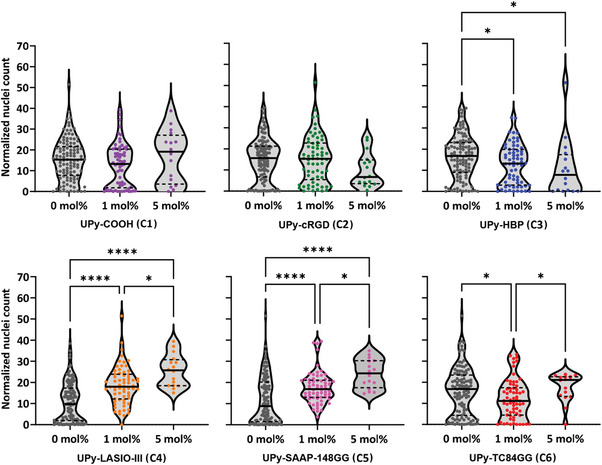
Cell adhesion of normal human dermal fibroblasts on microarray spots, grouped for each compound individually. The averaged normalized nuclei count was categorized for each compound into three groups; spots where the compound was absent (0 mol%), or present at 1 or 5 mol%. Statistical analyses showed a significant different between the indicated conditions with * *p* ≤ 0.05, ** *p* ≤ 0.01, *** *p* ≤ 0.001 and **** *p* ≤ 0.0001.

Next, a comparison was made between the different compounds for all spots, including mixtures of compounds. First, all spots were grouped for each individual compound for either the absence (0 mol%) or the presence (1 mol% or 5 mol%) of the compound, and statistically compared for significant differences (**Figure** **4**). Spots with 1 mol% of either the AMP UPy‐LASIO‐III (C4) or UPy‐SAAP‐148GG (C5) had significantly higher normalized cell counts compared to spots without either of these compounds, and even higher cell counts for their presence at 5 mol%. This same trend was observed for the spots where only C4 or C5 was present (Figure 3C). In contrast, presence of UPy‐HBP (C3) had a negative effect on the cell number for both 1 and 5 mol% compared to absence of C3. Finally, spots with 1 mol% of UPy‐TC84GG (C6) had a significantly lower normalized nuclei count compared to spots without C6 and spots with 5 mol% of C6. Neither the presence of UPy‐COOH (C1) or UPy‐cRGD (C2) had significant effects on the cell adhesion. The slightly enhanced trend observed for the single chemistry spots C2(5%) compared to C2(1%) as listed in Figure 3C, was lost when all spots with mixtures of C2 were taken into account. This result is surprising since NHDFs are known to bind to RGD via the *α*v*β*3 integrin on its cell membrane.^[^ ^44^, ^45^ ^]^ However, the lower cell adhesion may be explained by the lower local concentration of C2 at the material spots’ surface as a result from casting from DMSO instead of HFIP (Figure S1, Supporting Information).

Overall this data demonstrated that no cells adhered to the non‐functionalized surfaces and that the presence of only C4 or C5, or a mixture of either C4 or C5 with other compounds, significantly increased the cell count, whereas mixtures with C3 decreased the number of adhered cells.

To facilitate individual comparison of the effect of a combination of two compounds on the cell adhesion, a heatmap was created (Figure S4, Supporting Information). This revealed that presence of the AMP UPy‐LASIO‐III (C4) or UPy‐SAAP‐148GG (C5) in mixtures of compounds led to the highest nuclei counts. Presence of 1 mol% of C5 in mixtures, resulted in higher cell counts compared to presence of 1 mol% of C4. Also 5 mol% of the control UPy‐TC84GG (C6) caused an increase in cell adherence. Cell counts for any combination of the other components (C1, C2 or C3) remained low.

**Figure 5:**
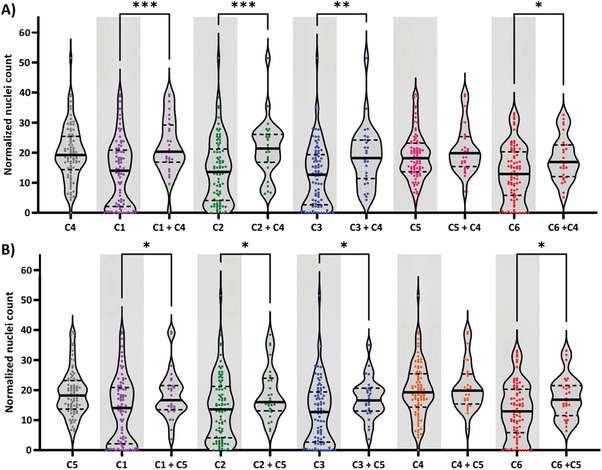
Effects of combining the AMP UPy‐LASIO‐III (C4) or UPy‐SAAP‐148GG (C5) with a second UPy‐additive. Violin plots of the averaged normalized nuclei count categorized per compound or as combination of two compounds. A) Combinations with C4 compared to the single compounds, both concentrations of 1 and 5 mol% were included. B) Combinations with C5 compared to the single compounds, both concentrations of 1 mol% and 5 mol% were included. Statistical analyses showed a significant different between the indicated conditions with * *p* ≤ 0.05, ** *p* ≤ 0.01, and *** *p* ≤ 0.001.

Violin plots were created to visualize possible synergistic effects in cell adhesion in combinations of either one of the AMPs C4 or C5 with C1, C2, C3 and C6. Here, results of spots with either 1 or 5 mol% were presented together (**Figure** **5**).

For the AMP UPy‐LASIO‐III (C4) as well as UPy‐SAAP‐148GG (C5) the same pattern in the was observed. A combination of the components C1, C2, C3 or C6 with C4 or C5, resulted in a significant increase in adhering cells compared to the absence of C4 or C5. However, no significant difference was observed when the combinations were compared to samples containing only C4 or only C5, which means that there was no significant enhanced or reduced cell adhesion upon addition of another additive to either C4 or C5. This includes the addition of a third additive (Figure S5, Supporting Information). These results are not surprising since there is a biological limit to the number of cells that can adhere to a spot. Spots with C4 and C5 present were already fully covered, leaving no room to evaluate synergistic effects in enhanced cell adhesion.

Another limitation of the system is that antimicrobial activity could not be evaluated. The slides could not be inoculated with a bacterial suspension to observe the effects of individual spots since the bacteria would grow in the medium covering the spots, thus obscuring any surface bactericidal activity. However, since the AMPs UPy‐LASIO‐III (C4)^[^ ^32^ ^]^ as well as UPy‐SAAP‐148GG^[^ ^38^ ^]^ are strongly bactericidal when applied to surfaces, the C4 and C5 containing coatings in this study most likely will be bactericidal as well.

## 3 Conclusion 

A supramolecular polymer microarray was fabricated for high throughput screening. The library, consisting of 3 replicates of 194 material spots, was printed on pHEMA coated glass slides via automated contact printing. Quality assessment with ToF‐SIMS confirmed that the compounds and spots were printed in the correct and designed pattern, showing it is a promising way to fabricate large libraries of supramolecular materials. Cell adhesion was assessed with NHDFs and was enhanced on the spots which contained at least the AMP UPy‐LASIO‐III, UPy‐SAAP‐148GG or a combination of both. The cell adhesion showed a concentration dependent effect, with higher number of adhering cells for 5 mol% compared to 1 mol%. No significant differences in cell adhesion between spots with combinations of UPy‐additives was observed. Overall it can be concluded that both AMPs UPy‐LASIO‐III and UPy‐SAAP‐148GG clearly favored cell adhesion. Apparently, only one UPy‐additive has to be incorporated in the material for two desired biological effects, cell adhesion and antimicrobial activity.^[^ ^32^, ^33^, ^34^, ^35^, ^38^ ^]^ Overall this study showed that supramolecular chemistry is eminently suitable for high throughput screening approaches due to the modular fashion in which different biological additives can be introduced and screened for a biological response.

## Conflict of Interest

The authors declare no conflict of interest.

## Supporting information

Supporting Information

## Data Availability

The data that support the findings of this study are available from the corresponding author upon reasonable request.
