# Supplementary material for: Merging Modular Molecular Design with High Throughput Screening of Cell Adhesion on Antimicrobial Supramolecular Biomaterials
Source: Macromol Rapid Commun. 2024 Apr 13;46(8):2300638. doi: 10.1002/marc.202300638 (PMC12004893; doi:10.1002/marc.202300638)
Supplement: Supplementary file 1 — Supporting Information [file MARC-46-2300638-s001.pdf]

Macromolecular Rapid Communications

Supporting Information

for *Macromol. Rapid Commun.*, DOI 10.1002/marc.202300638

Merging Modular Molecular Design with High Throughput Screening of Cell Adhesion on Antimicrobial Supramolecular Biomaterials

*Moniek G. J. Schmitz, Jasper G. M. Aarts, Laurence Burroughs, Phanikrishna Sudarsanam, Tim J. M. Kuijpers, Martijn Riool, Leonie de Boer, Xuan Xue, Dragan Bosnacki, Sebastian A. J. Zaat, Jan de Boer, Morgan R. Alexander and Patricia Y. W. Dankers**

Supporting Information

Merging modular molecular design with high throughput screening of antimicrobial supramolecular biomaterials

*Moniek G.J. Schmitz, Jasper G.M. Aarts, Laurence Burroughs, Phanikrishna Sudarsanam, Tim J.M. Kuijpers, Martijn Riool, Leonie de Boer, Xuan Xue, Dragan Bosnacki, Sebastian A.J. Zaat, Jan de Boer, Morgan R. Alexander, Patricia Y.W. Dankers**

Materials and Methods

Synthesis of various compounds/materials

PCLdiUPy[1], UPy-COOH[2], UPy-cRGD[3] (UPy-cyclic(RGDfK)) and UPy-LASIO-III (UPy-VNWKKILGKIIKVVK)[4] were synthesized by SyMO-Chem BV (Eindhoven, The Netherlands), as described previously.

Synthesis of SAAP-148GG and TC84GG

SAAP-148[5] and TC84GG[6] were synthesized by automated Fmoc-based solid phase peptide synthesis (SPPS). An extra glycine was introduced on both the C- and N-terminus of the AMPs, resulting in the amino acid sequences: GLKRVWKRVFKLLKRYWRQLKKPVRG and is referred to as SAAP-148GG and GLRAMCIKWWSGKHPKG and is referred to as TC84GG. The peptides were synthesized on a Fmoc-Sieber-TentaGel resin (Iris Biotech), 0.19 mmol/g in batches of 10 µmol scale with dimethylformamide (DMF) as solvent. As coupling system 2-(1H-benzotriazol-1-yl)-1,1,3,3 tetramethyluronium hexafluorophosphate (HBTU) in combination with N,N-diisopropylethylamine (DIPEA) was used in 1:1:4 molar equivalents of amino acid:HBTU:DIPEA. The amino acids (Novabiochem) were added in 4 times excess compared to the resin. A drop of Triton-X100 (Millipore) was added to each amino acid solution to prevent peptide aggregation. All couplings were performed in duplicate for 30 min. Fmoc deprotection was achieved with a 20% (v/v) solution of piperidine in DMF, 2 times for 10 min. After each coupling a capping step with pyridine:acetic anhydride:DMF (1:1:3 v/v/v) solution was performed for 8 min. The resin was washed with DMF in between all steps. After coupling of the last amino acid, the Fmoc was deprotected, yielding a peptide with a free amine N-terminus. Subsequently, the SAAP-148GG and TC84GG peptides were checked for successful synthesis with reversed phase high pressure

liquid chromatography mass spectrometry (RP-HPLC-MS) with a reverse phase C18 column and a linear gradient of 5-95% acetonitrile (ACN)/H_2O with 0.1% trifluoroacetic acid (TFA). After successful synthesis, 40 µmol of the SAAP-148GG batch was cleaved from the resin with 92.5% TFA, 2.5% triisopropylsilane (TIS), 2.5% D_2O and 2.5% 1,2-ethaneditihol (EDT) (v/v) and 80 µmol of the TC84GG batch was cleaved from the resin with 90% TFA, 2.5% TIS, 2.5% D_2O and 5% (v/v), for 3 h at room temperature (RT). The rest of the synthesis batch was used for coupling with the UPy-COOH synthon. After cleavage, the TFA was evaporated using a nitrogen-flow after which the cleaved peptide was precipitated in ice-cold diethylether, centrifuged at 2600 rpm for 5 min, dissolved in ACN/H_2O (1:6, v/v) and lyophilized. After filtration, the peptides were purified with a RP-HPLC-MS system and reverse phase C18 column with a linear gradient of 34-39% (SAAP-148GG) or 25-30% (TC84GG) ACN/H_2O with 0.1% TFA. All fractions were collected and lyophilized again. Subsequently, the lyophilized purified product was dissolved in 4 mM HCl to reduce the TFA counter ion and lyophilized once more resulting in an overall yield of 11.4% (15.2 mg, 4.5 µmol; SAAP-148GG) and 30.8% (48.1 mg, 24.6 µmol; TC84GG) with 95% and 96% purity, respectively. With Fluorine-19 nuclear magnetic resonance spectroscopy (^{19}F-NMR) on a Bruker UltraShield 400 MHz spectrometer, the residual presence of TFA was checked. The AMPs were dissolved at 4 mg/mL in D_2O and potassium hexafluoro phosphate was added as reference compound (4 mg/mL). Comparison of the integrals demonstrated that there was 0.004 or 2.2 TFA ion present per SAAP-148GG or TC84GG molecule, respectively. The purified AMPs were stored at -30 °C. ^{19}F NMR (376 MHz, D_2O, δ): 72.2 (d, KPF_6) integral 1.00, 75.6 (CF_3COOH) integral (SAAP-148GG = 0.00; TC84GG = 0.09). RP-HPLC-MS: calc exact mass = 3337.1 g/mol (SAAP-148GG), 1953.0 g/mol (TC84GG), found m/z for SAAP-148GG: 1669.5 $[M+2H]^{2+}$, 1113.9 $[M+3H]^{3+}$, 835.9 $[M+4H]^{4+}$ and for TC84: found m/z: 1954.2 $[M+H]^{+}$, 977.7 $[M+2H]^{2+}$, 652.1 $[M+3H]^{3+}$, 489.4 $[M+4H]^{4+}$.

Coupling of UPy - COOH to AMPs

After the peptide synthesis, the UPy-synthon was coupled to the deprotected N-terminus of the peptides. To 300 µmol of the SAAP-148GG batch and to 120 µmol of the TC84GG batch, an UPy-C_6-C_6-OEG_6-COOH linker was manually coupled in 2 equivalents to the N-terminus of the peptide (1 eq.) in one syringe. The UPy-coupling was performed while the peptide was still protected and, on the resin, using 1-[Bis(dimethylamino)methylene]-1H1,2,3-triazolo[4,5-b]pyridinium 3-Oxide Hexafluorophosphate (HATU; 2 eq.) and DIPEA (10 eq. (SAAP-148GG) or 5 eq. (TC84GG)) in DMF, on a shaker, at RT, overnight. Next, the UPy-

AMPs were deprotected and cleaved from the resin with the cleavage mixture of TFA/TIS/D_2O/EDT (95/2.5/2.5/2.5 v/v% (UPy-SAAP-148GG) or 90/2.5/2.5/5 v/v% (UPy-TC84GG)) for 3 h at RT. The TFA was removed with a flow of nitrogen and the UPy-peptide was precipitated in ice-cold diethylether. Next, the solution was centrifuged at 2600 rpm for 5 min and the pellet was dissolved in D_2O and lyophilized obtaining a white powder. After filtration the UPy-AMPs were purified on a RP-HPLC-MS system with a reverse phase C18 column, using a linear gradient of 40-45% (UPy-SAAP-148GG) or 33-38% (UPy-TC84GG) ACN/H_2O with 0.1% TFA. All fractions were collected and lyophilized. Subsequently, the same protocol as described before to remove the TFA counter ion was executed, and ^{19}F-NMR measurement revealed there were still 3.4 or 1.6 TFA ions present per UPy-SAAP-148GG or UPy-TC84GG molecule, respectively. The final product had a purity of >99% and was obtained in a yield of 8.5% (104.4 mg, 25.5 µmol; UPy-SAAP-148GG) and 27.7% (90.12 mg, 33 µmol; UPy-TC84GG). The purified UPy-AMPs were stored at -30 °C. ^{19}F NMR (376 MHz, D_2O, δ): 72.2 (d, KPF_6) integral 1.00, 75.6 (CF_3COOH) integral (UPy-SAAP-148GG = 0.07; UPy-TC84GG = 0.05). RP-HPLC-MS: calc exact mass = 4094.5 g/mol (UPy-SAAP-148GG); 2710.46 g/mol (UPy-TC84GG), found m/z for UPy-SAAP-148GG: 1366.2 $[M+3H]^{3+}$, 1025.1 $[M+4H]^{4+}$, 820.3 $[M+5H]^{5+}$, 683.8 $[M+6H]^{6+}$ and for UPy-TC84GG: 1356.2 $[M+2H]^{2+}$, 904.5 $[M+3H]^{3+}$, 678.6 $[M+4H]^{4+}$, 543.0 $[M+5H]^{5+}$, 453.0 $[M+6H]^{6+}$.

Synthesis of UPy-HBP (UPy-GLRKKLGKA)

The HBP (GLRKKLGKA) was synthesized by automated Fmoc-based SPPS. The peptide was synthesized on a Fmoc-Rink-Amide resin (Novabiochem), 0.51 mmol/g, 8 × 50 µmol scale in DMF. As coupling system HBTU in combination with DIPEA was used in 1:1:4 molar equivalents of amino acid:HBTU:DIPEA. The amino acids (Novabiochem) were added in 4 times excess compared to the resin. All couplings were performed in duplicate for 30 min. Fmoc deprotection was achieved with a 20% (v/v) solution of piperidine in DMF, 2 times for 10 min. After each coupling a capping step with pyridine:acetic anhydride:DMF (1:1:3 v/v/v) solution was performed for 8 min. The resin was washed with DMF in between all steps. After coupling of the last amino acid, the Fmoc was deprotected yielding a peptide with a free amine N-terminus. After the peptide synthesis all 8 batches were collected in one syringe to manually couple the UPy-COOH. The UPy-COOH (2 eq.) was coupled to the N-terminus of the peptide (1 eq.), while the peptide was still on the resin using HATU (2 eq.)

and DIPEA (10 eq.) in DMF at RT, for 22 h. Finally, the UPy-HBP was cleaved from the resin with 95% TFA, 2.5% TIS, 2.5% D_2O (v/v) for 3.5 h at RT. After cleavage, the TFA was evaporated using a nitrogen-flow after which the cleaved UPy-HBP was precipitated in ice-cold diethylether, centrifuged at 2600 rpm for 5 min and dissolved in ACN/H_2O (1:4 v/v) with 0.1% TFA. The cleaved UPy-HBP was purified using a preparative LC-ESI-MS system with a reverse phase C18 column. After filtration the UPy-HBP was purified using a linear gradient of 25-35% ACN/H_2O with 0.1% TFA. All fractions were collected and lyophilized. Subsequently, the UPy-HBP was dissolved in 4 mM HCl to reduce the TFA counter ion and lyophilized again resulting in a yield of 11.2% (77.6 mg, 45 µmol) with >99% purity. With ^{19}F-NMR, the residual presence of TFA was checked. UPy-HBP was dissolved at 4 mg/mL in D_2O and before the measurement potassium hexafluoro phosphate (4 mg/mL) was added as reference compound. Comparison of the integrals revealed that on average there were still 3.2 TFA ions present per UPy-HBP molecule. The purified UPy-HBP was stored at -30 °C. ^{19}F NMR (376 MHz, D_2O, δ): 72.2 (d, KPF_6) integral 1.00, 75.6 (CF_3COOH) integral 0.14. RP-HPLC-MS: calc exact mass = 1726.08 g/mol, found m/z: 1727.1 $[M+H]^+$, 864.0 $[M+2H]^{2+}$, 576.5 $[M+3H]^{3+}$, 432.7 $[M+4H]^{4+}$, 346.1 $[M+5H]^{5+}$.

Epoxy glass slide manufacturing

60 glass microscope slides (25 mm × 75 mm, Sigma) were placed into a metal slide rack and activated using a Diener Nano plasma system with O_2 gas supply (p = 0.9 mbar, 40 kHz, 1000 W, 5 min). They were immediately then transferred into dry (4 Å molecular sieves) toluene (500 mL) under argon. (3-Glycidyloxypropyl)trimethoxysilane (10 mL) was then added to the solution, and the reaction mixture heated to 50 °C for 3 h. The slides were then cooled to RT and washed by sonication with 2 x 100 mL fresh acetone. The slides were then dried under vacuum in a silicone-free Heraeus Vacuum Oven (35 °C, 0.3 mbar) for 24 h.

poly-HEMA Coated Slides

A 4% (w/v) poly-HEMA (Sigma, P3932 BioReagent Grade) in 95% (v/v) ethanol/H_2O solution was sonicated for 24 h at RT to ensure complete dissolution. Epoxy coated glass array slides were dip-coated in this poly-HEMA solution using a Holmarc HO-TH-01 dip-coater using a dip speed of 9 mm/s, retraction speed of 2 mm/s and dip duration of 2 s; the slides were allowed to dry for 10 min between dips and were dipped a total of 4 times. The slides were then allowed to dry for a further 16 h at ambient conditions, before being placed in a silicone-free Heraeus vacuum oven (35 °C, 0.3 mbar) for 48 h.

Design of the library

The library was designed with 6 different UPy-additives and the PCLdiUPy base polymer. In total 3 spots of solely base polymer PCLdiUPy were present in the library. Besides this, base polymer was mixed with the single additives at 1 or 5 mol% or with a combination of 2 or 3 additives. In the combination spots, the additives were present at 1 or 5 mol%, but 5 mol% was never incorporated more than once. This resulted in PCLdiUPy:UPy-additive ratios of 99:1, 98:2, 97:3, 95:5, 94:6, 93:7 and yielded in a library with 194 spots.

Preparation of master plate

A solution of PCLdiUPy was prepared at 7.1 mM (20 mg/mL) in DMSO. All UPy additives were prepared in stock solutions of 7.1 mM in DMSO. The solutions were manually pipetted into a 384 well plate in the correct ratios, 30 µL per well, to create the master plate and molecular library. PCLdiUPy:UPy-additive ratios of 99:1, 98:2, 97:3, 95:5, 94:6, 93:7 were obtained containing either, 1, 2, 3, 5, 6 or 7 mol% additive solutions.

Microarray fabrication

Polymer microarrays were printed on to poly-HEMA dip-coated slides and epoxy functionalized slides using a XYZ3200 dispensing station (Biodot) using 3 quilled silicon pins (Parallel Synthesis, SMT-S100 100 µm tip). Printing was carried out maintaining 60-65% relative humidity. Diluted polymer solutions were composed of base polymer PCLdiUPy at 20 mg/mL concentration in combination with additives at 1 mol% or 5 mol% as noted. Three replicates were printed on each slide. A total of 9 spots were printed per row; spacing between the printed spots in each row was 1500 µm in the x axis, with a 750 µm spacing between each row in the y axis and an alternating +750 µm/-750 µm offset in the x axis between each row. After printing, the microarrays were dried in a vacuum oven (35 °C, 0.3 mbar) for 24 h. The stability of the microarray spots was assessed by submerging the microarray in PBS (ThermoFisher, Gibco, pH = 7.4) for 2 weeks at 37 °C. Not all spots were the similar in size, but the spot size was independent of the chemistry **(Figure S3)**.

Time-of-Flight Secondary Ion Mass Spectrometry (ToF-SIMS) measurements

ToF-SIMS measurements were conducted using a ToF-SIMS IV (IONTOF GmbH) instrument operated using a 25 kV Bi_3^+ primary ion. The primary ion beam was rasterized over an analysis area of 13.75 × 17.5 mm to capture the entire array. Positive secondary ion spectra were collected. Owing to the non-conductive nature of the samples, a low energy (20 eV) electron flood gun was applied to provide charge compensation.

Brightfield microscopy

Images were captured using an automated stage microscope (IMSTAR) with phase contrast filter and 4× objective magnification.

Preparation of dropcast films

Solutions of PCLdiUPy were prepared at 7.1 mM (20 mg/mL) in HFIP or DMSO. UPy-cRGD was dissolved at the same concentration of 7.1 mM in HFIP or DMSO. These solutions were mixed in molar ratios of PCLdiUPy:UPy-cRGD of 95:5 obtaining 5 mol% UPy-cRGD samples. Dropcast films were prepared by casting 2 µL of the polymer solutions on a glass microscope slide. The polymer films on the glass coverslips were air-dried for 1 h before drying in vacuum overnight. The casting was performed in a relative humidity of around 40%.

Atomic force microscopy (AFM)

AFM was performed at RT using either a Digital Instrument Multimode Nanoscope IV or a Digital Instrument Dimension 3100 Nanoscope IIIa, operating in tapping regime mode using silicon cantilever tips (PPP-NCHR, NanoSensors, 204-497 kHz, 10-130 N/m). Height and phase images of the dropcast and dipcoated films were recorded in air. The images were processed with Gwyddion software (version 2.52).

Cell culture and staining on microarray

NHDF cells (Lonza) were used and cultured with DMEM (Gibco), supplemented with 10 % (v/v) FBS (Fisher biotec) and 10 U/mL of penicillin/streptomycin. Cells were grown at 37° C in an incubator at 5% CO_2. For the microarray experiments, cells at passage 4-5 were used at a seeding density of 10,000 cells/cm^2. Cells were treated with 10 ng/mL of TGF-β1 (Peprotech) in the media during the cell seeding on the microarray. The cells were incubated on the microarray for 48 h.

After cell culture, cells were washed with PBS) and fixed with 3.9 % (w/v) paraformaldehyde (Fisher) for 15 min at RT. Each washing step was performed with PBS three times unless mentioned different and all the steps were performed at RT. Cells were washed and permeabilized using 0.5 % Triton-X100 for 15 min followed by washing. Then the cells were blocked with 3 % bovine serum albumin (BSA; Roche) in PBS (w/v) for 30 min and washed and blocked for unspecific binding with 10 % goat serum (life technologies) in PBS for another 30 min. Afterwards, cells were incubated with the primary antibody, anti- alpha smooth muscle actin (1:600; Sigma Aldrich) in PBS and incubated at 4 °C overnight. Next day, cells were washed and incubated with fluorescently-tagged secondary antibody with an Alex flour 488 (1:500; Molecular probes) targeting the primary antibody and incubated for 1 h in the dark. After washing, cells were stained with phalloidin tetramethyl rhodamine (TRITC) (1:200; Sigma Aldrich) in PBS for 45 min and washed. Finally, cells were counterstained for nucleus with 4′,6-diamidino-2-phenylindole (DAPI) (1:500; Sigma) and washed with PBS. The stained microarray slide was then mounted with a glass coverslip and used for imaging.

Cell imaging – fluorescence microscopy

The stained microarray was inverted and used to acquire the images using an automated Nikon Eclipse Ti-2 microscope equipped with a Photometrics Prime 95 B camera.

CellProfiler analysis

Acquired fluorescent images were analyzed with CellProfiler 4.1.3 software[7] using a custom-made pipeline. The total nuclei count per sample was captured by the Otsu adaptive thresholding method applied on the DAPI image channel. Wrong segmentation artifacts were corrected by manually checking the individual images.

Determination of the spot size

Spot radii of all individual spots were determined using NIS Elements 5.21.02, using the radius 3-points circle measuring tool. The area was determined and the total nuclei count per sample was normalized using the following formula:

$$\text{Nuclei Count} = (\text{area}_{spot} / \text{area}_{max}) \times \text{nuclei count}_{spot}$$

where $area_{max}$ = spot with largest area, $area_{spot}$ = area of individual spot, and nuclei $count_{spot}$ = nuclei count for corresponding $area_{spot}$.

Statistical analysis

The data was checked for a normal distribution with the Kolmogorov-Smirnov test and all different data sets did not show a normal distribution. This was followed by one-way ANOVA Kruskal-Wallis and post-hoc uncorrected Dunnet's test using GraphPad Prism 9.0 software. The heatmaps were created with the R library package ggplot2 (ggplot2 version 3.3.5, R version 4.0.5)[8].

Supplementary figures

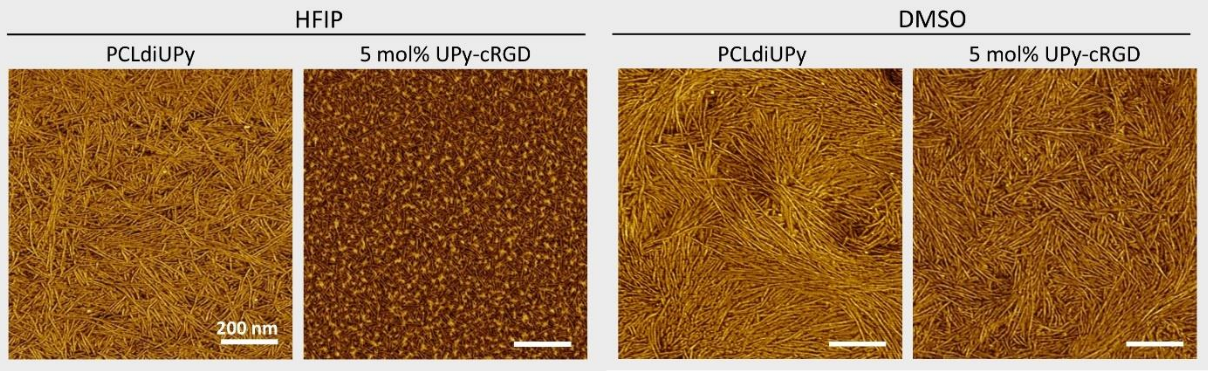

Figure S1. AFM phase images of different solution-cast films. Comparison of 2 µL dropcast films with PCLdiUPy without and with 5 mol% UPy-cRGD from HFIP and DMSO.

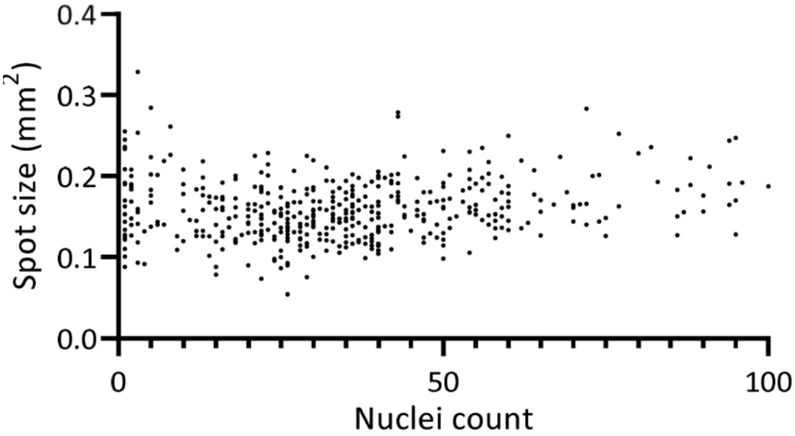

Figure S2. Normal human dermal fibroblast cell adhesion on microarray spots; correlation of the nuclei count versus the spot size.

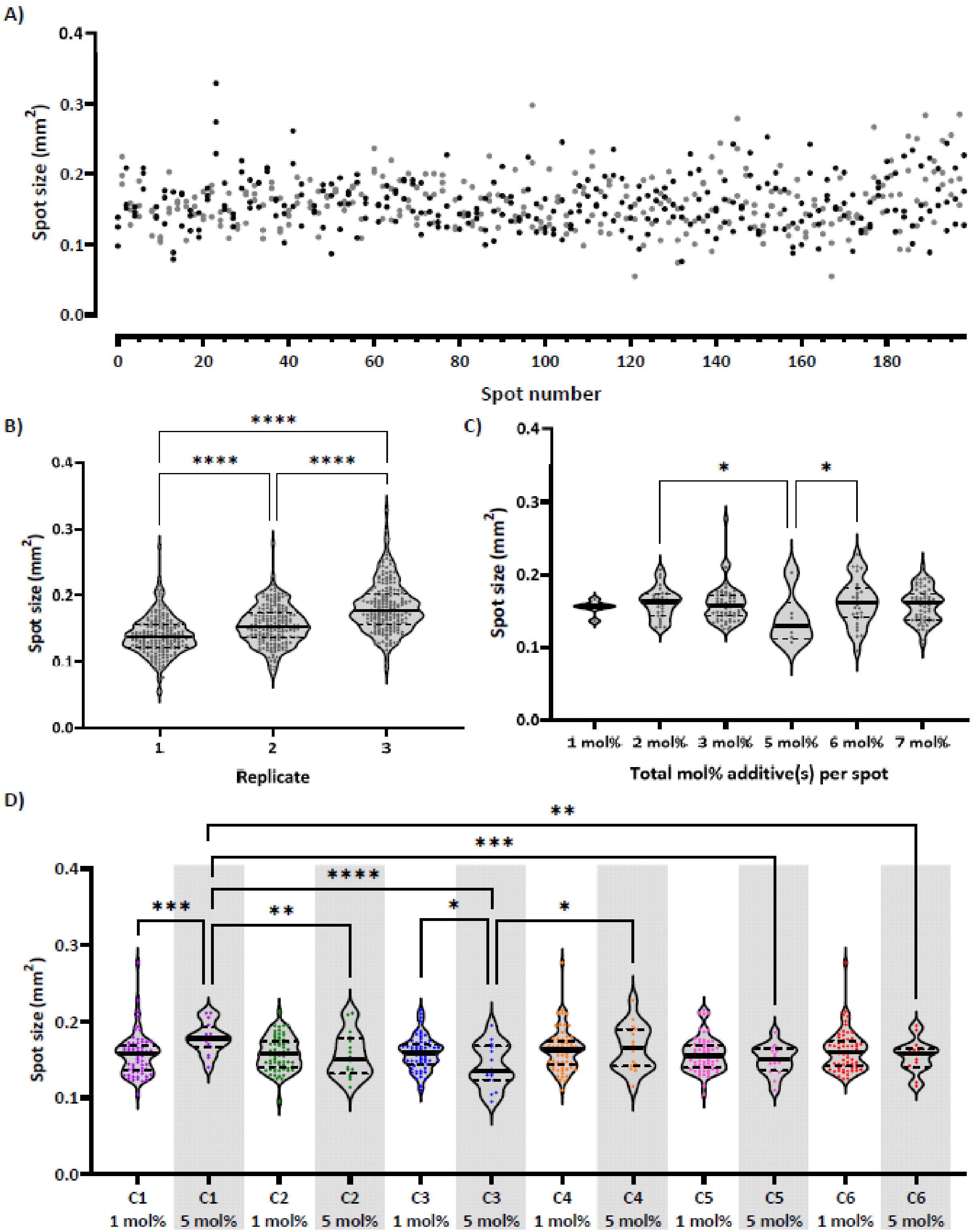

Figure S3. Correlation of microarray spot size to the technical replicate, total mol% UPy-additive and chemical composition. A) Plot of all the individual spot sizes. B) Spot sizes grouped per replicate. C) Spot sizes grouped by total mol% of UPy - additive(s) per spot. D) Spot sizes grouped by chemical composition, either 1 or 5 mol% of additives C1 - C6. Statistical analyses showed a significant different between the indicated conditions with * $p \leq 0.05$, ** $p \leq 0.01$, *** $p \leq 0.001$ and **** $p \leq 0.0001$.

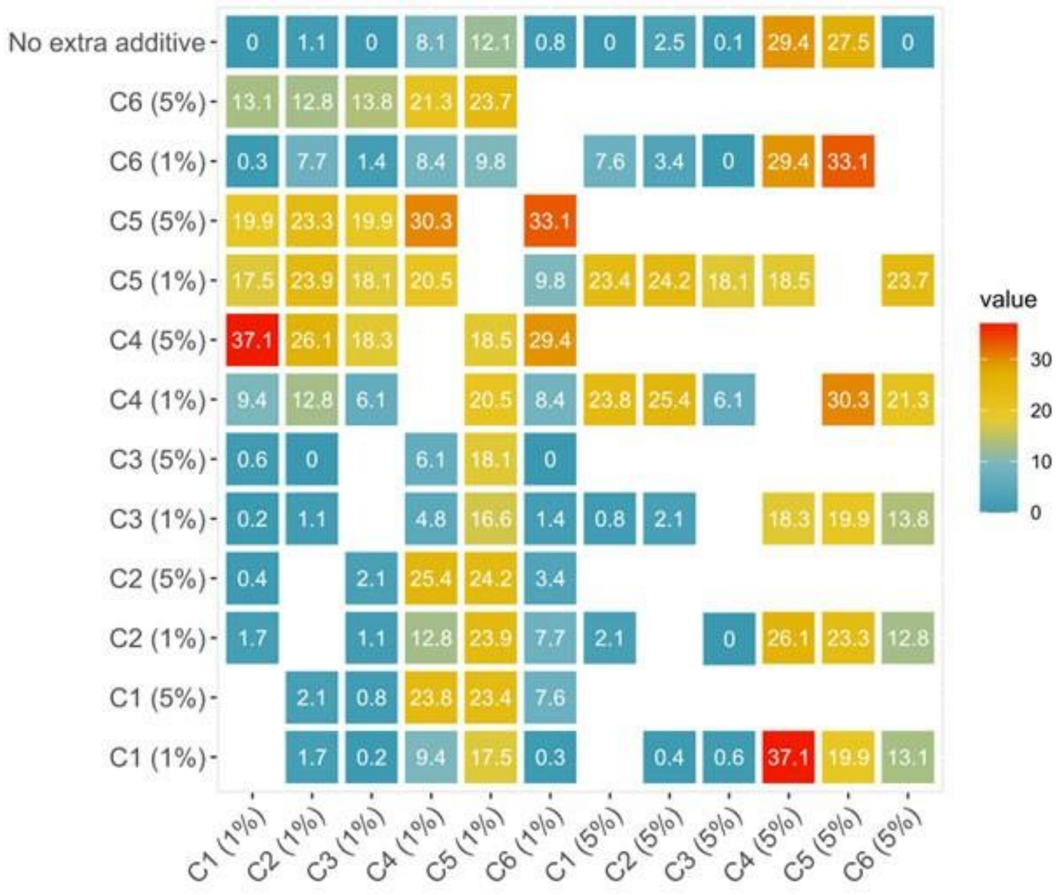

Figure S4. Heatmap showing the averaged normalized nuclei count for a combination of two UPy-additive compounds.

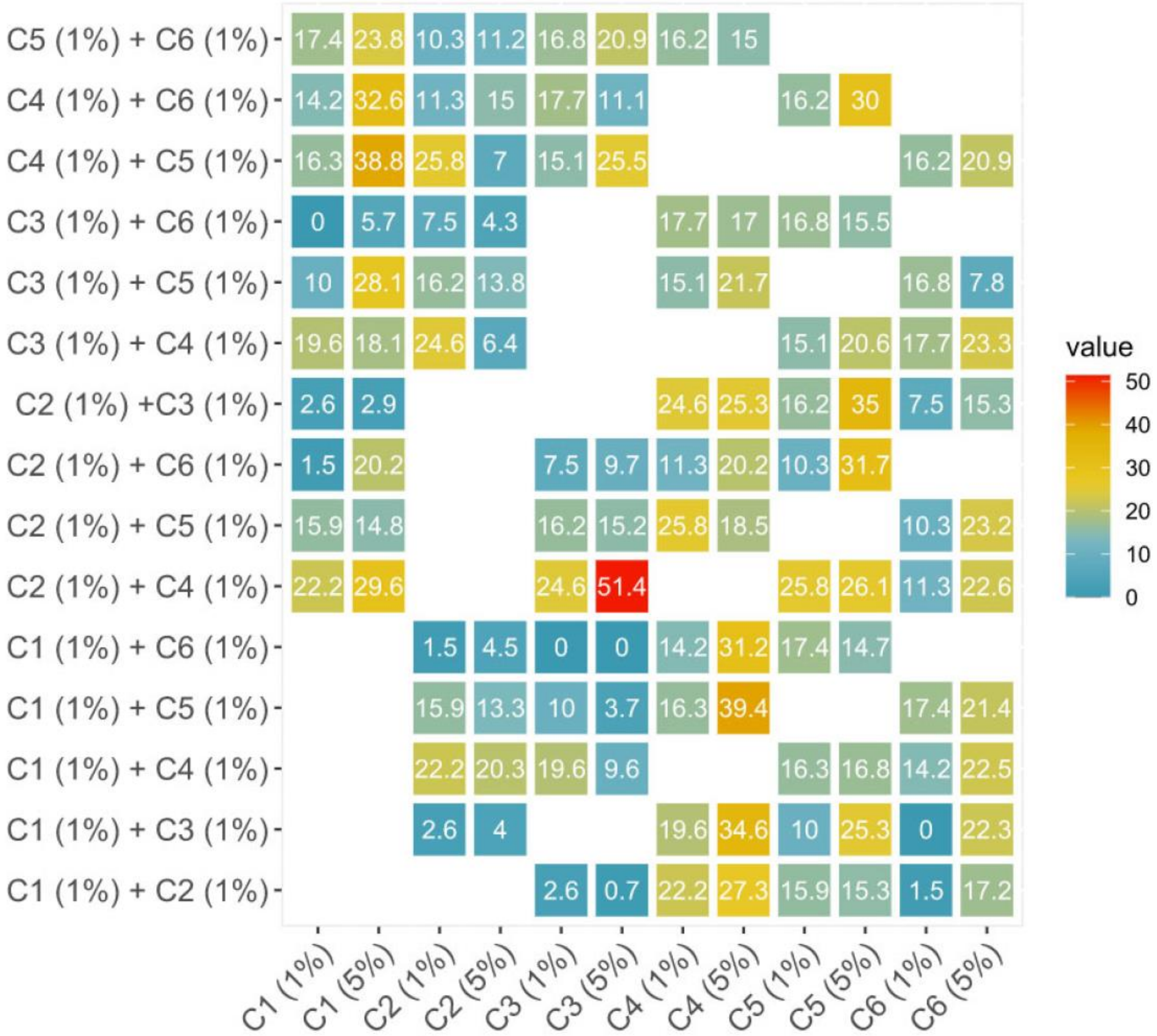

Figure S5. Heatmap showing the averaged normalized nuclei count for a combination of three UPy-additive compounds.

References

[1] B. B. Mollet, M. Comellas-Aragonès, A. J. H. Spiering, S. H. M. Söntjens, E. W. Meijer, P. Y. W. Dankers, *J. Mater. Chem. B.* **2014**, *2*, 2321.

[2] I. De Feijter, O. J. G. M. Goor, S. I. S. Hendrikse, M. Comellas - Aragonès, S. H. M. Söntjens, S. Zaccaria, P. P. K. H. Fransen, J. W. Peeters, L. G. Milroy, P. Y. W. Dankers, *Synlett* **2015**, *26*, 2707.

[3] R. C. van Gaal, A. B. C. Buskermolen, B. D. Ippel, P. P. K. H. Fransen, S. Zaccaria, C. V. C. Bouten, P. Y. W. Dankers, *Biomaterials* **2019**, *224*, 119466.

[4] S. Zaccaria, R. C. Van Gaal, M. Riool, S. A. J. Zaat, P. Y. W. Dankers, *J. Polym. Sci. Part A Polym. Chem.* **2018**, *56*, 1926.

[5] A. de Breij, M. Riool, R. A. Cordfunke, N. Malanovic, L. de Boer, R. I. Koning, E. Ravensbergen, M. Franken, T. van der Heijde, B. K. Boekema, et al., *Sci. Transl. Med.* **2018**, *10*, eaan4044.

[6] S. Omardien, J. W. Drijfhout, H. van Veen, S. Schachtschabel, M. Riool, L. W. Hamoen, S. Brul, S. A. J. Zaat, *Biochim. Biophys. Acta - Biomembr.* **2018**, *1860*, 2416–2427.

[7] C. McQuin, A. Goodman, V. Chernyshev, L. Kamentsky, B. A. Cimini, K. W. Karhohs, M. Doan, L. Ding, S. M. Rafelski, D. Thirstrup, et al. *PLoS Biol.* **2018**, *16*, 1–17.

[8] Wickham, H. **2016**. Ggplot2: Elegant graphics for data analysis (2nd ed.) Springer International Publishing.
